# 33rd Annual Meeting & Pre-Conference Programs of the Society for Immunotherapy of Cancer (SITC 2018): Late-Breaking Abstracts: Washington, D.C., USA. 7-11 November 2018

**DOI:** 10.1186/s40425-018-0434-7

**Published:** 2018-12-06

## O48 In silico assessment of variation in TMB quantification across diagnostic platforms: Phase 1 of the Friends of Cancer Research Harmonization Project

### David Fabrizio, PhD^1^, Shu-Jen Chen, PhD^2^, Mingchao Xie, PhD^3^, Wangjuh (Sting) Chen, PhD^4^, Katie J. Quinn, PhD^5^, Chen Zhao, PhD^6^, Ahmet Zehir, PhD^7^, Vincent Funari, PhD (CGMBS)^8^, Jennifer S. Dickey, PhD^9^, Vikas Gupta, PhD^10^, Dinesh Cyanam, PhD^11^, Lisa M. McShane, PhD^12^, Naiyer A. Rizvi, MD^13^, Matthew Hellmann, MD^7^, Mark Stewart, PhD^14^, Diana M. Merino, PhD^14^, Jeff Allen, PhD^14^, Friends of Cancer Research TMB Harmonization Team^14^

#### ^1^Foundation Medicine, Cambridge, MA, USA; ^2^ACT Genomics, Taipei City, Taiwan; ^3^AstraZeneca, Waltham, MA, USA; ^4^Caris Life Sciences, Phoenix, AZ, USA; ^5^Guardant Health, Redwood City, CA, USA; ^6^Illumina, San Diego, CA, USA; ^7^Memorial Sloan Kettering Cancer Center, New York, NY, USA; ^8^NeoGenomics Laboratories, Inc., Aliso Viego, CA, USA; ^9^Personal Genome Diagnotics, Baltimore, MD, USA; ^10^Qiagen, Aarhus, Denmark; ^11^Thermo Fisher Scientific, Ann Arbor, MI, USA; ^12^National Cancer Institute, Bethesda, MD, USA; ^13^Columbia University Medical Center, New York, NY, USA; ^14^Friends of Cancer Research, Washington, DC, USA

##### **Correspondence:** Jeff Allen (jallen@focr.org)

**Background**

Tumor mutational burden (TMB) is a measure of the number of somatic mutations and a predictive biomarker of response to immune checkpoint inhibitors (ICI) across several cancers. TMB can be estimated using targeted next-generation sequencing (NGS), but differences in quantification can arise based on platform differences, testing panel size and composition, and bioinformatic algorithms. Harmonization of methods to quantify TMB will facilitate biomarker development and optimize clinical utilization and treatment decision-making. Friends of Cancer Research (Friends) convened a group of leading diagnostic partners to assess and identify sources of TMB variability and determine best practices for harmonizing TMB estimation to ensure consistent clinical interpretation in the future.

**Method**

Eleven diagnostic members of the Friends TMB Harmonization Team used whole exome sequencing (WES) data from The Cancer Genome Atlas (TCGA) MC3 samples, comprising 32 cancer types. Each diagnostic partner calculated TMB from the subset of the exome restricted to the genes covered by their targeted panel and using their own bioinformatics pipeline (panel-derived TMB). A “gold-standard” TMB estimate was calculated from the entire exome using a uniform bioinformatics pipeline that all members agreed upon (WES-derived TMB). Linear regression analyses were performed to investigate relationship between WES-derived TMB and each panel-derived TMB. Exploratory analyses by cancer type were also performed. Bias and variability in TMB estimates across panel-derived TMB values were assessed.

**Results**

In silico quantification of TMB is relatively consistent between panels across a wide range of TMB values (0-40 mut/Mb). Panel-derived TMB strongly correlated with WES-derived TMB (regression R2 values range across panels 0.85-0.93, with slopes ranging from 0.82-1.37). Variation in TMB quantification was attributable to unique composition and technical specifications of each panel, as well as differences in the underlying algorithms used to estimate TMB from observed somatic mutations. Exploratory analyses suggested possible cancer type dependence for the relationship of panel vs WES-derived TMB, meriting further investigation.

**Conclusions**

In this in silico analysis, panel-derived TMB was strongly correlated with WES-derived TMB. Some variation in TMB quantification across panel-based diagnostic platforms exists. Identifying factors that contribute to variation will facilitate harmonization and help ensure appropriate use and implementation of tests results in the clinic. Subsequent steps will assess the effect of biologic factors (e.g. specimen type, cancer type, treatment setting), the impact of variation on clinical outcomes, align standards, and define best practices for quantification of TMB.

## O49 Imaging of tumor infiltrating T cells with an anti-CD8 minibody 89Zr-IAB22M2C in advanced solid tumors: a phase I first-in-human study

### Michael S. Gordon, MD^1^, Frank Tsai^1^, Michael Postow, MD^2^, Matthew Hellmann, MD^2^, James J. Harding, MD^2^, Ronald L. Korn, MD^3^, Michael D. Farwell, MD^4^, Tara C. Mitchell^4^, Lynn M. Schuchter, MD^4^, Martha Ziolkowska^2^, Joseph O'Donoghue^2^, Jason S. Lewis, ^2^ Anna M. Wu, PhD^5^, William Le^6^, Ian Wilson^6^, Wolfgang A. Weber^2^, Jedd D. Wolchok, MD, PhD^2^, Deepak Behera, MD^6^, Neeta Pandit-Taskar^2^

#### ^1^HonorHealth Research Institute, Scottsdale, AZ, USA; ^2^Memorial Sloan Kettering Cancer Center, New York, NY, USA; ^3^Imaging Endpoints, Scottsdale, AZ, USA; ^4^University of Pennsylvania, Philadelphia, PA, USA; ^5^City of Hope, Duarte, CA, USA; ^6^ImaginAb Inc, Inglewood, CA, USA

##### **Correspondence:** Deepak Behera (dbehera@imaginab.com)

**Background**

The degree and character of tumor infiltration by CD8 T-cells is associated with favorable outcomes to immunotherapy. Biopsies to assess T-cell infiltration are invasive, and one biopsy may not capture the immunologic heterogeneity that exists among various tumors in an individual patient. Non-invasive CD8 T-cell imaging could provide a more comprehensive view of T-cell infiltration and potentially correlate with patient outcomes.

**Method**

We conducted a phase 1 first-in-human study assessing the ability of positron emission tomography (PET) scans using anti-CD8 radiolabeled minibody, 89Zr-IAB22M2C (CD8-tracer), to detect whole body and tumor CD8 distribution, in patients with metastatic solid tumors. All patients received 3mCi 89Zr-IAB22M2C once intravenously followed by serial PET scans over a 5-7 day period. The study was conducted in two stages. During stage 1, 6 patients received increasing protein doses, 0.2 mg through 10 mg (dose escalation), to establish safety and determine appropriate scanning parameters. In Stage 2 (dose expansion) an additional 9 subjects were scanned to better delineate our recommended phase 2 dose. All patients were monitored for drug-related adverse events and evaluated with blood chemistry, hematology, cytokine assay and anti-drug antibodies (ADA). Biodistribution, radiodosimetry and semi-quantitative evaluation of CD8-tracer uptake were performed in all patients.

**Results**

15 Subjects with metastatic cancer were enrolled (31-82 years, M/F = 9/6). Primary cancer types were melanoma (n=8), non-small cell lung carcinoma (n=6), and hepatocellular carcinoma (n=1). Two subjects were treatment naïve, 3 had discontinued prior treatment, and 10 were on immunotherapy for 2 weeks to >2 years. There were no drug-related adverse reactions, cytokine release or blood test abnormalities. Transient increase in ADA was noted in 1/15 subject. The CD8-tracer rapidly cleared from the blood and accumulated in CD8 rich tissues (e.g. spleen, bone marrow and lymph nodes), which were saturable as demonstrated by decreasing activity with increasing protein dose (Figure 1). Tracer uptake in tumors was variable and noted in 10/15 subjects as early as 1-2hr post-infusion (Figure 2). Tracer excretion was primarily hepato-biliary. Low background was seen in non-T cell rich tissues such as muscle, brain and heart. Biodistribution and pharmacokinetics were favorable between 0.5-1.5 mg protein doses. The estimated mean effective radiation dose was 24 rem/mCi.

**Conclusions**

Our first-in-human study demonstrated the safety of the CD8-tracer 89Zr-IAB22M2C and provided detailed whole-body information demonstrating the biodistribution of CD8 T-cells in tumors and reference tissues with the possibility of same-day imaging.

**Acknowledgements**

Research Support: ImaginAb, Inc., Parker Institute for Cancer Immunotherapy

**Ethics Approval**

The study was approved by Institutional Review Boards of MSKCC (IRB #16-1109), Honor Health (West IRB #1179278) and University of Pennsylvania (IRB # 828992).

**Consent**

Written informed consent was obtained from the patient for publication of this abstract and any accompanying images. A copy of the written consent is available for review by the Editor of this journal.

**Trial Registration**

ClinicalTrials.gov Identifier: NCT03107663

**Fig. 1 (abstract O49).**
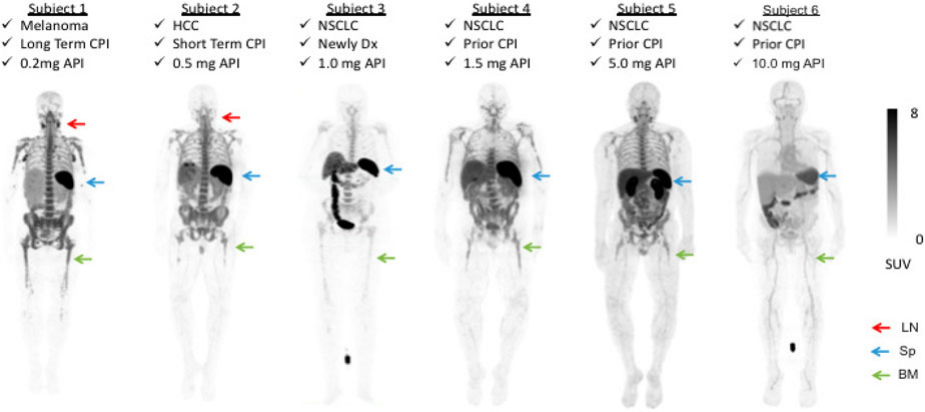
89Zr-IAB22M2C PET images in dose escalation stage

**Fig. 2 (abstract O49).**
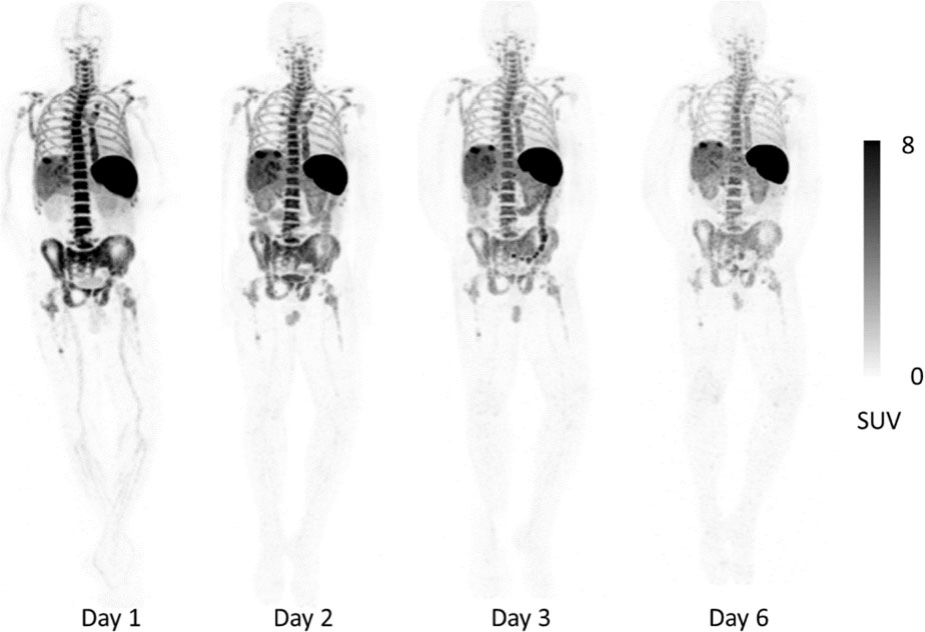
Stable 89Zr-IAB22M2C uptake over time

## O50 Pembrolizumab in combination with chemoradiotherapy (CRT) in human papilloma virus (HPV)-associated head and neck squamous cell carcinoma (HNSCC)

### Steven F. Powell, MD^1^, Mark Gitau, MD^2^, John Reynolds, MD^1^, Andrew Terrell, MD^2^, Michele M. Lohr, MD, MS^1^, Steven McGraw, MD^1^, Ryan K. Nowak, MD^1^, Ash Jensen, MD^2^, Miran Blanchard, MD^2^, Christopher D. Fischer, MD^3^, Christie Ellison, RN^4^, Lora Black, MPH^4^, Paul A. Thompson, PhD^4^, Kathryn Gold, MD^5^, Ezra Cohen, MD^5^, Julie Bykowski, MD^5^, John Lee, MD^6^, William C. Spanos, MD^1^

#### ^1^Sanford Cancer Center, Sioux Falls, SD, USA; ^2^Roger Maris Cancer Center, Fargo, ND, USA; ^3^Sanford Clinic Molecular Imaging, Sioux Falls, SD, USA; ^4^Sanford Research, Sioux Falls, SD, USA; ^5^Moores Cancer Center UCSD, San Diego, CA, USA; ^6^Chan Soon-Shiong Institute for Medicine, Culver City, CA, USA

##### **Correspondence:** William C. Spanos (william.spanos@sanfordhealth.org)

**Background**

Inhibitors of the programmed death receptor-1 (PD-1) and its ligand (PD-L1) have activity in recurrent and metastatic HNSCC. Now these agents are moving rapidly into curative-intent treatment approaches. Our group has previously reported the safety of adding the PD-1 inhibitor, pembrolizumab, to concurrent cisplatin-based CRT [1]. Here we will report the efficacy of this approach in HPV-associated HNSCC.

**Method**

Patients (pts) with stage III-IVB HPV-associated (based on p16 immunohistochemistry) HNSCC eligible for definitive CRT were enrolled as part of an expansion cohort. The treatment regimen is outlined in Figure 1 (Figure 1). Efficacy was the primary endpoint defined as complete response (CR) rate on imaging or with salvage surgery (pathologic CR) at 100 days post-CRT completion. Imaging CR was determined by RECIST 1.1 criteria for computed tomography (CT) and/or Hopkins Criteria (Score 1, 2 or 3) [2] for positron emission tomography (PET). Progression-free survival (PFS), overall survival (OS), and locoregional control (LRC) rate were key secondary endpoints.

**Results**

From November 2015 to February 2018, 34 pts with HPV-associated HNSCC were enrolled and assessable for the primary endpoint with median follow-up of 21 months (range 8-26 months). Demographic and disease characteristics are outlined in Table 1 (Table 1). 29 (85%) of pts achieved a CR based on imaging and/or surgical criteria. An additional 2 pts were felt to have no clinical evidence of disease despite imaging findings and did not undergo salvage surgery. 1 (2.9%) patient developed progressive disease with distant metastases [3]. PFS at 1 year was 97.1% (95% CI 80.9% - 99.6%). Treatment compliance is outlined in Table 2 (Table 2). Acute CRT-associated grade ≥3 toxicities included mucositis (n=12, 35%), dysphagia (n=21, 62%), and radiation dermatitis (n=2, 6%).

**Conclusions**

The addition of pembrolizumab to low-dose cisplatin-based CRT in a predominantly intermediate-risk HPV+ population displayed promising response and early PFS data. No new immune-related safety signals were seen in this expansion cohort. Several phase III trials further evaluating the efficacy of this approach are underway.

**Acknowledgements**

We would like to acknowledge the Merck Investigator Studies Program for providing grant support for this study.

**Ethics Approval**

The study was approved by the WIRB Institutional Review Board, approval number 20152167.

**Trial Registration**

NCT02586207

**References**

1. Powell SF, Gitau MM, Sumey CJ, Reynolds JT, Lohr M, McGraw S, Nowak RK, Terrell AM, Jensen AW, Blanchard MJ, Ellison C, Black LJ, Thompson PA, Gold KA, Cohen EEW, Lee JH, Spanos WC. Safety of pembrolizumab with chemoradiation (CRT) in locally advanced squamous cell carcinoma of the head and neck (LA-SCCHN). J Clin Oncol 35, 2017 (suppl, abstr 6011)

2. Marcus C, Ciarallo A, Tahari AK, Mena E, Koch W, Wahl RL, Kiess AP, Kang H, Subramaniam RM. Head and neck PET/CT: therapy response interpretation criteria (Hopkins Criteria)-interreader reliability, accuracy, and survival outcomes. J Nucl Med. 2014 Sep;55(9):1411-6.

3. O'Sullivan B, Huang SH, Siu LL, Waldron J, Zhao H, Perez-Ordonez B, Weinreb I, Kim J, Ringash J, Bayley A, Dawson LA, Hope A, Cho J, Irish J, Gilbert R, Gullane P, Hui A, Liu FF, Chen E, Xu W., Deintensification candidate subgroups in human papillomavirus-related oropharyngeal cancer according to minimal risk of distant metastasis. J Clin Oncol, 2013. Feb 10;31(5):543-50.

**Fig. 1 (abstract O50).**
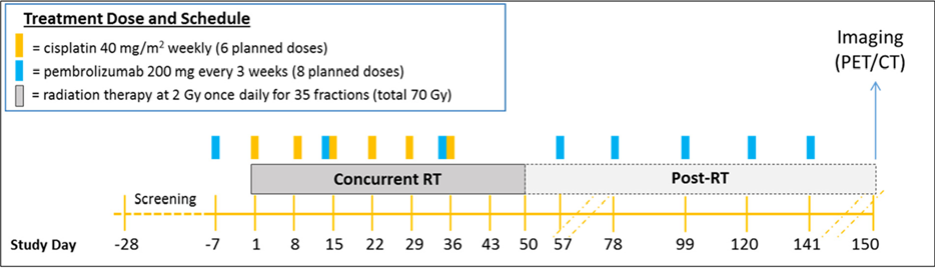
Study Treatment

**Table 1 (abstract O50).**
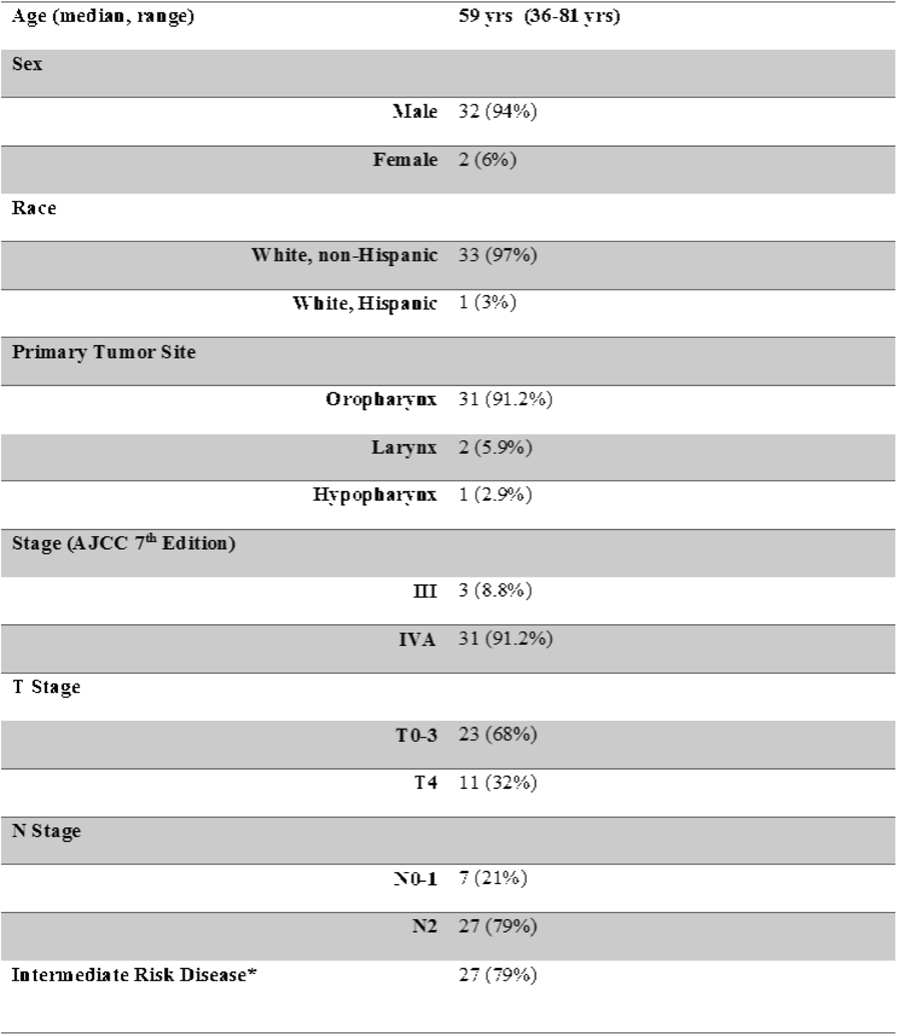
Demographics and Disease Characteristics

**Table 2 (abstract O50).**
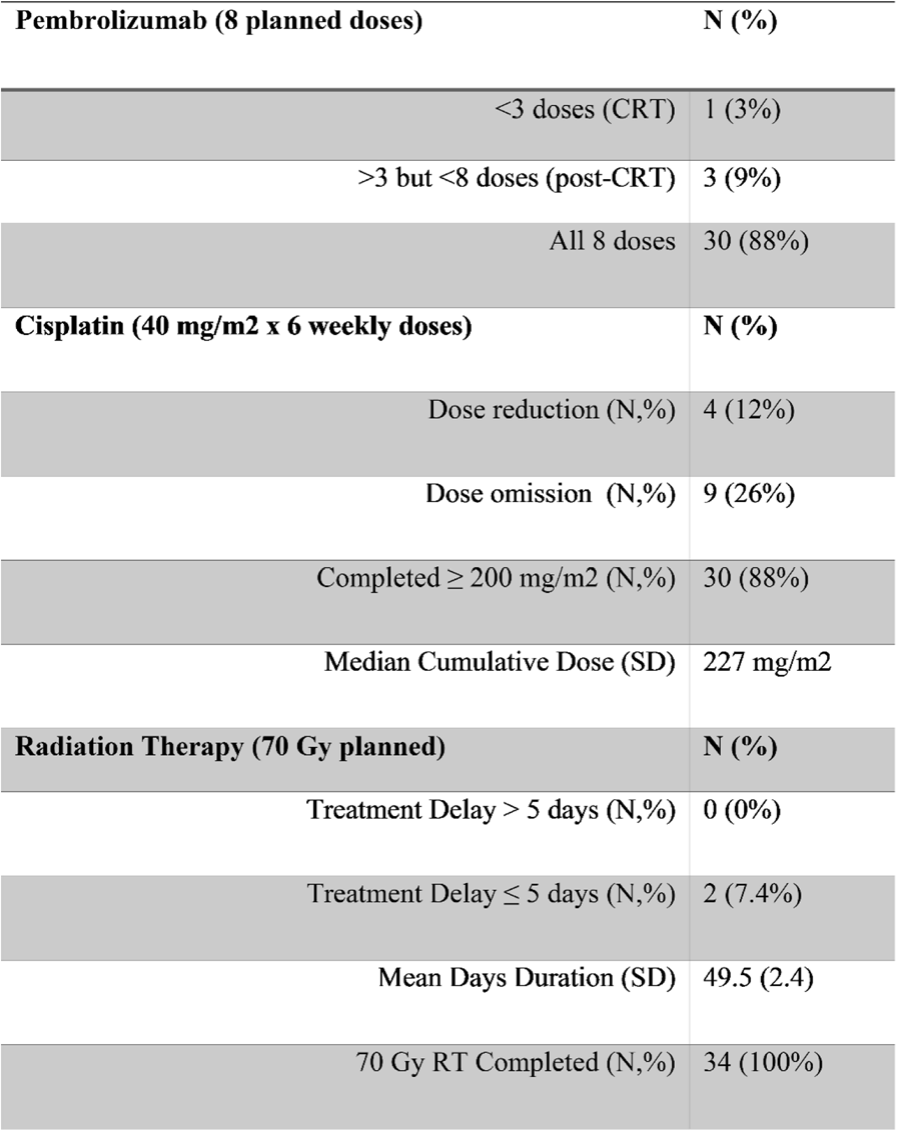
Treatment Compliance

## O51 Monalizumab in combination with cetuximab in recurrent or metastatic squamous cell carcinoma of the head and neck (R/M SCCHN): clinical and translational biomarker results.

### Roger Cohen^1^, Jerome Fayette^2^, Marshall Posner, MD^3^, Gautier Lefebvre^4^, Jessica Bauman, MD^5^, Sebastien Salas^6^, Caroline Even^7^, Dimitrios Colevas^8^, Antonio Jimeno, MD, PhD^9^, Esma Saada-Bouzid, MD, PhD^10^, Barbara A. Burtness, MD^11^, Franceline Calmels^12^, Robert Zerbib, MSc^12^, Agnès Boyer-Chammard, MD^12^, Pascale André, PhD^12^, Tanguy Seiwert, MD^13^

#### ^1^Abramson Cancer Center, Philadelphia, PA, USA; ^2^Centre Leon Berard, Lyon, France; ^3^Mount Sinai Medical Center, New York, NY, USA; ^4^Oscar Lambret Institute, Lille, France; ^5^Fox Case Cancer Center, Philadelphia, PA, USA; ^6^AP-HM, Marseille, France; ^7^Institut Gustave Roussy, Villejuif, France; ^8^Stanford University Medial Center, Stanford, CA, USA; ^9^University of Colorado, Denver, CO, USA; ^10^Centre Antoine Lacassagne, Nice, France; ^11^Yale University, New Haven, CT, USA; ^12^Innate Pharma, Marseille, France; ^13^University of Chicago, Chicago, IL, USA

##### **Correspondence:** Roger Cohen (roger.cohen@uphs.upenn.edu)

**Background**

Monalizumab is a novel immune checkpoint inhibitor targeting NKG2A (Natural Killer Group 2A), which is expressed as a heterodimer with CD94 on subsets of NK (Natural Killer) cells, T cells and tumor-infiltrating CD8+ T cells. The NKG2A ligand, HLA-E, is upregulated in cancer, including SCCHN. NKG2A blockade promotes innate anti-tumor immunity mediated by NK and CD8+ T cells and enhances human NK cell antibody-dependent cell-mediated cytotoxicity (ADCC) induced by cetuximab. Cetuximab is approved for SCCHN patients (pts) progressing after platinum-based chemotherapy, with a response rate of 13%.

**Method**

This is a multicenter phase I/II trial testing the combination of monalizumab and cetuximab in pts with advanced SCCHN. The phase I part was previously reported showing a favorable safety profile for the combination. In the phase II part, pts received monalizumab at the recommended phase 2 dose of 10 mg/kg q2weeks and cetuximab according to the label (loading dose 400 then 250 mg/m2 q1week). Pts were required to be progressing after platinum-based chemotherapy and to have received <2 prior lines of therapy in the R/M setting. The primary endpoint was ORR per RECIST assessed every 8 weeks. Pts were treated until disease progression or unacceptable toxicity. Various biomarkers were assessed in tumor biopsies and peripheral blood.

**Results**

As of August 31, 2018, 40 pts have been enrolled and are evaluable for safety and efficacy. All pts received prior platinum-based chemotherapy, 17 (43%) prior anti-PD-(L)1 and 5 (13%), prior cetuximab. The most frequent related adverse events (AEs) were fatigue (18%), rash (15%) and hypophosphatemia (15%). Most AEs were Grade 1-2, 18% of pts had treatment-related grade 3-4 AEs. ORR was 27.5% [16.1-42.8], with 11 confirmed responses (1 complete and 10 partial responses). With a median follow-up of 8 months, median duration of response, PFS and OS were 5.0 months [3.7-6.9] and 10.3 months [7.3-NR], respectively. Data show a trend towards an increase in infiltrating NKp46+ and CD8+ cells in available biopsies of responders after first administration. Additional biomarker analyses will be presented (including tumor mutation burden, HLA-E and NKG2A expression on peripheral and tumor infiltrating lymphocytes).

**Conclusions**

These data confirm the activity and safety of monalizumab in combination with cetuximab in R/M SCCHN, with deep and durable responses. This study continues to enroll additional R/M SCCHN patients who received both platinum-based chemotherapy and PD-(L) 1 inhibitors.

**Trial Registration**

NCT02643550

## O52 Intratumoral injection of a novel oncolytic virus, Voyager V1 (VV1): completed phase 1 monotherapy in patients with refractory solid tumors

### Steven F. Powell, MD^1^, Jaime Merchan^2^, Manish R. Patel, DO^3^, Timothy P. Cripe, MD, PhD^4^, James Strauss, MD^5^, Matthew Old, MD^6^, Rosa M. Diaz, PhD^7^, Kah Whye Peng, PhD^7^, Stephen J. Russell, MD, PhD^7^, Alice S. Bexon^7^, Steven F. Powell, MD^1^

#### ^1^Sanford Health, Sioux Falls, SD, USA; ^2^U Miami, Miami, FL, USA; ^3^U Minnesota, Minneapolis, MD, USA; ^4^Nationwide Children’s, Columbus, OH, USA; ^5^Mary Crowley Cancer Center, Dallas, TX, USA; ^6^Ohio State University, Columbus, OH, USA; ^7^Vyriad, Rochester, MN, USA

##### **Correspondence:** Alice S. Bexon (abexon@vyriad.com)

**Background**

VV1 is derived from VSV, an RNA virus with low human seroprevalence, engineered to replicate selectively in and kill human cancer cells. VV1 encodes hIFNβ gene to boost antitumoral immune response, plus the thyroidal sodium iodide symporter NIS gene to allow noninvasive imaging of virus spread. VV1 is synergistic with different anti-PD-(L)1 antibodies in preclinical models. Here we report first-in-human data from the completed IT monotherapy dose escalation.

**Method**

This was an open label phase 1 dose escalation study with a classical 3+3 design. Single VV1 doses from 3 x 106 to 3 x I09 TCID50 were injected IT into one target lesion. Dose increments were ½ log. Objectives include identifying the monotherapy MTD and RP2D, preliminary efficacy, PK by RT-PCR for viral genomes, serum IFNβ levels, and Tc-99m SPECT/CT imaging.

**Results**

The study is ongoing at dose level 6 (n=20). No DLTs have been observed. Most pts were male (55%), white (85%), with ECOG PS median 1 (range 0-1) and median 5 lines of prior systemic therapy for head & neck (SCCHN, 30%), colon (25%), rectal (5%), pancreas (15%), breast (10%), lung (5%) or other (10%) cancers. AEs (in 80% pts) related to VV1 were mild-moderate, short-lived clinical AEs or transient G2-4 neutro- or lymphopenia at higher doses. More pts had related AEs at higher doses, including a G2 cytokine release syndrome and G1 hypotension (both SAEs). Ten pts had mild biopsy-or injection-related AEs (pain, swelling, bruising, subclinical pneumothorax), and two had an SAE related to procedure (pneumothorax). There were no deaths related to treatment or procedure. Seven pts at higher doses had positive SPECT/CTs to date, showing viral replication in tumor +/- concomitant lymphocyte/neutrophil trafficking. Most pts at higher dose levels had IFNß in serum indicative of viral replication in tumor, but no positive viral titers or shedding. IFNβ levels show a dose response relationship. One pancreas cancer pt had tumor cavitation with cystic fluid positive for viral RNA and IFNß. Five pts had stable disease on CT at D43, two with decrease in sum of target lesions.

**Conclusions**

IT Voyager-V1 is well-tolerated as a single agent. Injection reactions are manageable with few serious AEs or toxicity >G2. There are indications of antitumor efficacy, and evidence of viral replication based on observed disease stability with positive SPECT and detectable IFNß at higher doses. Data from the highest planned dose level will be reported at presentation.

## P706 Effects of indoximod plus gemcitabine/nab-paclitaxel on tumor microenvironment of patients with metastatic pancreas cancer

### Jiayi Yu, PhD^1^, Gabriela R. Rossi, PhD^1^, Devora Delman^2^, Joey Li, BS^2^, Ravindra Kolhe, MD, PhD^3^, David H. Munn, MD^3^, Nathan Bahary, MD, PhD^4^, Nicholas Vahanian, MD^1^, Eugene P. Kennedy, MD, FACS^1^, Gregory L. Beatty, MD, PhD^2^, Charles Link, Jr., MD^1^

#### ^1^NewLink Genetics, Ankeny, IA, USA; ^2^University of Pennsylvania, Philadelphia, PA, USA; ^3^Medical College of Georgia, Augusta Univ, Augusta, GA, USA; ^4^Hillman Cancer Center, Pittsburgh, PA, USA

##### **Correspondence:** Gabriela R. Rossi (gabyrossi@icloud.com)

**Background**

The indoleamine 2,3-dioxygenase (IDO) pathway mediates immunosuppressive effects through the metabolism of tryptophan (Trp) to kynurenine (Kyn). This metabolic pathway triggers downstream signaling through the Trp sensors GCN2 and mTOR and the Kyn sensor AHR [1-4]. Indoximod is an orally administered, small-molecule IDO pathway inhibitor that reverses the immunosuppressive effects of low Trp and high Kyn that result from IDO activity. The mechanism of action (MOA) of indoximod targets four main cell types: CD8+ T cells, CD4+ helper and regulatory T cells, and DCs. Indoximod has immunostimulatory effects by increasing proliferation of effector T cells, reprograming Treg into helper T cells, and downregulation of IDO expression in DCs. These effects are observed in both the presence and absence of IDO activity [5].

**Method**

Treatment-naïve metastatic pancreas cancer patients were treated with combination of indoximod and gemcitabine/nab-paclitaxel (SOC) in Phase 2 trial (NCT02077881). Patients underwent pre-treatment tumor biopsy with a repeat biopsy on week 8. Sixteen pairs of tumor specimens (8 patients with objective response, and 8 non-responders) underwent RNA sequencing analysis and multiplex immunohistochemistry (IHC) staining to assess the phenotype and functional status of multiple immune populations in the TME.

**Results**

Upon treatment, significantly higher intratumoral CD3+ (p = 0.04) and CD8+ T (p = 0.003) cells density were observed in clinical-responding patients compared to non-responding patients. For these tumor-infiltrated CD3+ cells, higher T cell proliferation (Ki67+) and T cell function (GzmB+) were observed in responding patients. Higher GzmB in CD3- cells (most likely NK cells) was also detected in responding patients. Additionally, CD4+ T cells, NK cells, macrophages and neutrophils were induced in these patients upon treatment. Importantly, consistent with indoximod MOA, both Tregs (Foxp3+) population (p = 0.05) and IDO1 expression (p = 0.04) were significantly reduced after treatment. Moreover, the CD8: Foxp3 T-cell ratio (p = 0.0008) was significantly increased. These findings provided strong evidences to support the hypothesized MOA for indoximod [5] including the upregulation of both innate and adaptive immune responses in TME.

**Conclusions**

The treatment with indoximod plus SOC induced increased density and activity of intratumoral T cells, increased activity of innate immune cells (NK cells) in responding patients. This treatment also significantly downregulated Treg population and IDO expression in the TME. The combination of indoximod and SOC increased both innate and adaptive immune responses in TME of patients with metastatic pancreas cancer in strong support with the proposed MOA for indoximod.

**Trial Registration**

ClinicalTrials.gov Identifier NCT02077881

**References**

1. McGaha TL, et al. Immunol Rev. 2012;249(1):135-157.

2. Munn DH, et al. Immunity. 2005;22(5):633-642.

3. Metz R, et al. OncoImmunology. 2012;1(9):1460-1468.

4. Opitz CA, et al. Nature. 2011;478(7368):197-203.

5. Brincks EL, et al. Presented at: Annual Meeting of the American Association for Cancer Research (AACR), April 14-18, 2018; Chicago, IL. Abstract 3753.

## P707 Tumor mutational burden (TMB) ring study: Comparison of multiple targeted next-generation sequencing (NGS) sequencing platforms

### Jeffrey Conroy, BS^1^, Sarabjot Pabla, MSc, PhD, BS^1^, Yirong Wang, MS^1^, Sean T. Glenn, PhD^1^, Razelle Kurzrock, MD^2^, Shumei Kato, MD^2^, Ryosuke Okamura^2^, Denis A. Smirnov^3^, Brad Foulk^3^, Traci Pawlowski, PhD^4^, Dinesh Cyanam, PhD^5^, Geoffrey M. Lowman, PhD^5^, Blake Burgher, BS, RN^1^, Jacob Hagen^1^, Mary Nesline, MS^1^, Antonios Papanicolau-Sengos, MD^1^, Felicia L. Lenzo^1^, Mark Gardner^1^, Carl D. Morrison, MD, DVM^1^

#### ^1^OmniSeq, Inc., Buffalo, NY, USA; ^2^UC San Diego Moores Cancer Center, San Diego, CA, USA; ^3^Janssen Research and Development, Sping Hous, PA, USA; ^4^Illumina, Inc., San Diego, CA, USA; ^5^ThermoFisherScientific, Carlsbad, CA, USA

##### **Correspondence:** Carl D. Morrison (carl.morrison@omniseq.com)

**Background**

Tumor mutational burden (TMB), a measurement of the frequency of mutations in tumor cells, is currently being evaluated as a biomarker to predict response to immune checkpoint inhibitors. Whole exome sequencing is considered the gold standard assay, but is inefficient and too costly to run routinely. Consequently, several targeted NGS assays have been designed to measure TMB. In this study, we compared TMB measurements from four targeted NGS assays using a common source of specimens. Concordance and accuracy of TMB values, cutoffs and clinical interpretations were assessed.

**Method**

Genomic DNA from 161 FFPE specimens representing 24 tumor types was extracted following anatomical pathologist review. TMB testing was completed or first attempted by Foundation Medicine (FoundationOne®), followed by on-site analysis by OmniSeq (Immune Report Card®), Illumina (TruSight Oncology 500™), and ThermoFisher (Oncomine™ Tumor Mutation Load) from a subsequent central DNA isolation. Each laboratory followed its own protocol for reporting TMB values (mutations/Mb). Pairwise Pearson product-moment correlations (R) were performed to estimate concordance of TMB values between platforms. 150 gold standards were established (7 TMB-high, 143 TMB-low) for which at least three of four platforms were concordant when using a TMB-high cutoff of ≥10. Each platform was assessed for TMB interpretation accuracy at this threshold.

**Results**

TMB values were successfully reported for >90% of samples and were concordant across platforms (R = 0.74 - 0.91). TMB distribution for each platform demonstrated a value of 10 mutations/Mb within the 84th-91st percentiles and captured all consensus TMB-high gold standard samples at >90% accuracy. Where results were available, the seven TMB-high gold standard samples were identified across all platforms resulting in 100% sensitivity. For the 143 TMB-low, the number of false positives ranged from 2-11, resulting in 89.6-98.6% specificity. PPV ranged from 31.3-77.8% across all tumor types, but improved to ≥75% across platforms when restricted to NSCLC samples (n=14). Pair-wise linear regression model fits did not significantly improve concordance between platforms (p>0.05).

**Conclusions**

The TMB assays evaluated were robust across a wide range of solid tumor specimens. There is general concordance between the platforms despite considerable variability in TMB calling parameters and the genes targeted. Nevertheless, each platform is highly accurate when using a TMB-high cutoff of ≥10, which improves when restricted to NSCLC. This study suggests that TMB can be measured accurately across multiple platforms, but further studies utilizing additional NGS platforms and samples are required to validate these findings.

**Ethics Approval**

OmniSeq’s analysis utilized deidentified data that qualified as non-human subject research under IRB protocol (BDR #080316) approved by Roswell Park Comprehensive Cancer Center (Buffalo, NY).

## P708 Immunopharmacodynamic responses of Imprime PGG combined with Pembrolizumab in chemotherapy-resistant metastatic triple negative breast cancer subjects in a Phase 2 trial: Analyses of Stage 1 patients

### Nadine Ottoson, BS^1^, Adria B. Jonas, MS^2^, Anissa S. Chan, PhD^2^, Xiaohong Qiu, BS^2^, Blaine Rathmann, BS^2^, Richard Walsh, BS^1^, Ben Harrison, MS^2^, Mike Danielson, PhD^2^, Kyle S. Michel, BA^2^, Michaela Finley^2^, Mark Uhlik, PhD^2^, Jamie Lowe, BA^2^, Paulette Mattson, BFA^2^, Michele A. Gargano, MS^2^, Michael J. Chisamore, PhD^3^, Joanna Cox, MD^2^, Bruno Osterwalder, MD^4^, Jeremy R. Graff, PhD^2^, Nandita Bose, PhD, Nadine Ottoson, BS^1^

#### ^1^Biothera Pharmaceuticals, Inc., Eagan, MN, USA; ^2^Biothera, Eagan, MN, USA; ^3^Merck & Co, Rahway, NJ, USA; ^4^B.O. Consulting GmbH, Riehen, Switzerland

##### **Correspondence:** Nadine Ottoson (nottoson@biothera.com)

**Background**

Though efficacious, checkpoint inhibitor (CPI) monotherapy fails to elicit response in the majority of patients. TNBC is one such cancer type where CPI antibodies (pembrolizumab, avelumab, atezolizumab), have demonstrated only a ~5-10% response rate, irrespective of PD-L1 expression. We are developing Imprime PGG (Imprime), a novel yeast derived β-glucan PAMP in combination with pembrolizumab, to enhance the benefit that TNBC patients derive from CPI-based therapy.

**Method**

In this analysis, we present the serum and cellular IPD responses elicited by Imprime and pembrolizumab in the peripheral blood of 12 TNBC subjects who previously failed front-line chemotherapy, enrolled as part of a Phase 2 study (NCT02981303). Subjects received Imprime (4 mg/kg qw) + Pembro IV (200 mg q3w) in 3 week cycles. Anti-beta glucan antibodies (ABA), circulating immune complexes (CIC), complement activation, cytokine production, gene expression changes, and phenotypic changes on immune cells were evaluated.

**Results**

As Imprime is known to complex with serum IgG ABA, a drop in the free ABA levels and a concomitant increase in the CIC was observed at the end of infusion (EOI) of every Imprime dose. Interestingly, 11 of 12 subjects showed increased ABA levels between cycles 1 and 2, with peak levels increasing ~1.5 to 35-fold over baseline. In line with this ABA increase, peak levels in serum CIC levels (range ~3 to 22-fold) and complement protein SC5b-9 (~1.4 to 41-fold) were also observed at cycle 2 EOI. In a subset of patients, a maximum increase of ~10-1000-fold in several chemokines was detected at cycle 2 EOI. Gene expression analyses of whole blood indicated peak activation of several genes at cycle 2 associated with activation of innate immune cells and T-cells. In 8 of 12 subjects, an increased frequency up to 11-fold in the CD16+ monocytes, cells known for their enhanced cytotoxicity as well as M1-polarizing functions, was observed between cycles 1 and 2. We also observed an increase, up to 2-fold, in CD16+ inflammatory DC in 8 of 12 subjects. The maximal increase (~4 to 20-fold) in newly proliferating (Ki67+), activated CD8 T cells (PD-1+ CD38+ HLADR+) was observed at cycle 2 in 4 subjects. Of all these immunological responses, robust cytokine production together with an increased frequency of activated CD8 T cells, correlated with objective tumor responses.

**Conclusions**

These data provide the first evidence in cancer patients that Imprime can drive the critical IPD changes known to be associated with efficacy in preclinical cancer models.

**Ethics Approval**

The study was approved by central IRB (Western Institutional Review Board) under approval number 20162506 for sites that are able to use a central IRB, for sites with local IRBs, approval was obtained for each site by their respective IRB for this study.

**Consent**

Written informed consent was obtained from the patient for publication of this abstract and accompanying images. A copy of the written consent is available for review

**Trial Registration**

NCT02981303

## P709 Peripheral Blood TCRB Chain Convergence Predicts Response to Dendritic Cell-Based Immunotherapy in Advanced-Stage Melanoma Patients

### Walter J. Storkus, PhD^1^, Yan Lin^1^, Lauren Miller, BS^2^, Denise S. Topacio-Hall, BS, MA^2^, Geoffrey M. Lowman, PhD^2^, Timothy Looney, PhD^2^, Lisa H. Butterfield, PhD^1^, Jennifer L. Taylor, PhD^1^, Ahmad A. Tarhini, MD, PhD^3^, Hussein A. Tawbi, MD, PhD^4^, John M. Kirkwood, MD^1^

#### ^1^University of Pittsburgh, Pittsburgh, PA, USA; ^2^ThermoFisher, Carlsbad, CA, USA; ^3^Cleveland Clinic Foundation, Cleveland, OH, USA; ^4^MD Anderson Cancer Center, Houston, TX, USA

##### **Correspondence:** John M. Kirkwood (kirkwoodjm@upmc.edu)

**Background**

T cell receptor (TCR) convergence refers to the phenomenon whereby antigen-driven selection enriches for TCRs having a shared antigen specificity but different nucleotide sequences. TCR convergence may be indicative of tumor immunogenicity and thus the sensitivity of a cancer to immunotherapy. Here we used next-generation sequencing of peripheral blood TCRB chain repertoires to evaluate TCR convergence as a predictive biomarker for response to dendritic cell-based immunotherapy for advanced melanoma. We further evaluated the relationship between TCR convergence and response biomarkers derived from targeted gene expression profiling of pre-treatment tumor biopsies.

**Method**

Total RNA was extracted from peripheral blood leukocytes (PBL) isolated from 13 evaluable HLA-A2+ patients with advanced-stage melanoma treated with dasatinib plus an autologous dendritic cell/peptide-based vaccine targeting 6 tumor-associated vascular antigens (UPCI 12-048, NCT01876212), which included 6 responders (4 PR, 2 SD) and 7 non-responders (PD). TCRB chain repertoire libraries were constructed by multiplex PCR utilizing FR1 and constant gene targeting primers via the Oncomine TCRB-LR assay, then sequenced using the Ion Torrent S5 to a target depth of 1.5M raw reads per library. To evaluate T cell repertoire convergence we searched for instances where TCRB chains were identical in amino acid space but had distinct nucleotide sequences owing to N-addition and exonucleotide chewback within the V-D and D-J junctions of the CDR3. Targeted gene expression profiling of pre- and post-treatment tumor biopsies was performed via the Oncomine Immune Response Research Assay using total RNA input.

**Results**

Sequencing of TCRB libraries yielded on average 20k clonotypes per individual with mean evenness (normalized Shannon entropy) of .84. TCR convergence was elevated in pretreatment PBL of responders compared to non-responders (mean frequency .012 vs .006, p=.01, Wilcoxon), discriminated responders from non-responders with high accuracy (AUROC = .90), and closely correlated with time to progression following treatment (Spearman correlation = .78). Targeted gene expression profiling of tumor revealed elevated PD-L1 expression in pre-treatment responders compared to non-responders and reduced PTP1B and HIF1A in post-treatment biopsies from responders compared to non-responders. Combining pre-treatment PBL TCR convergence values with tumor PD-L1 expression values improved the prediction of response.

**Conclusions**

These data suggest that peripheral blood TCRB convergence may serve as a biomarker for response to dendritic cell-based immunotherapy, to be used alone or in combination with established biomarkers derived from profiling of the tumor microenvironment. Ongoing and future studies will further clarify the prognostic and/or predictive utility of this immune repertoire biomarker.

**Acknowledgements**

This work was supported by NIH R01 CA168118 (WJS).

**Ethics Approval**

This study was approved by the University of Pittsburgh's IRB, approval PRO12060479.

## P710 Phase 1/2 study of image guided intratumoral CD40 agonistic monoclonal antibody (APX005M) in combination with systemic pembrolizumab for treatment-naïve metastatic melanoma

### Salah Eddine Bentebibel^1^, Daniel H. Johnson, MD^1^, Srisuda Lecagoonporn, PhD DPharm^1^, Cara Haymaker, PhD^1^, Houssein Safa, MD^1^, Cassian Yee, MD^1^, Rodabe N. Amaria, MD^1^, Sapna Patel, MD^1^, Hussein A. Tawbi, MD, PhD^1^, Isabella C. Glitza, MD, PhD^1^, Michael Davies, MD, PhD^1^, Michael K. Wong, MD PhD FRCPC^1^, Wen-Jen Hwu, MD, PhD^1^, Patrick Hwu, MD^1^, Willem W. Overwijk, PhD^1^, Ovid C. Trifan, MD, PhD^2^, Chantale Bernatchez^1^, Adi Diab, MD^1^

#### ^1^MD Anderson Cancer Center, Houston, TX, USA; ^2^Apexigen Inc., San Carlos, CA, USA

##### **Correspondence:** Adi Diab (adiab@mdanderson.org)

**Background**

Immune-checkpoint blockade has become a major modality in the treatment of metastatic melanoma. However, long-term survival and durable remission rates remain low and new treatment options are needed to improve clinical outcome. CD40 activation on antigen presenting cells (APCs) initiates their ability to prime and activate CD8+ T cells through upregulation of co-stimulatory molecules as well as expression of effector cytokines. APX005M is a humanized IgG1 CD40 agonistic antibody that binds with high affinity to human CD40 expressed on APCs. Our pre-clinical studies have demonstrated that intratumoral (IT) CD40 activation induced systemic anti-tumor effects and augmented the activity of anti-PD-1. We hypothesized that this combination will stimulate local APCs resulting in activation of tumor-specific CD8+ T cells and distant responses

**Method**

This is an ongoing dose escalation and expansion study of image guided IT CD40 agonistic monoclonal antibody (APX005M) in combination with standard systemic pembrolizumab in metastatic melanoma with an accelerated 3+3 design. Approximately 36 participants will be enrolled, all patients (pts) will receive IT APX005M every 3 weeks for a total of 4 doses. The primary objectives of the study are to evaluate safety and tolerability, determine the recommended phase 2 dose (RP2D), and assess the overall response rate (ORR) 12 weeks after treatment initiation by RECIST 1.1 at RP2D. The dose escalation portion of the trial has enrolled 10 pts in 5 dose escalating cohorts of APX005M at 0.1, 0.5, 1, 3 and 10 mg in combination with standard pembrolizumab at 2 mg/Kg. 26 pts will have 75% power to detect an improvement from a null ORR of 33% to 55%, using a one group chi-square test and assuming a one-sided α level of 5%. Biomarker analyses of blood and tumor biopsies both in injected and non-injected tumors are being performed to measure immune activation using immunophenotyping including Mass Cytometry (CyTOF), Multiplexed ion beam imaging analysis (MIBI), TCR sequencing and gene expression analyses.

**Results**

9 pts treated across all five dosing cohorts have had disease evaluations (as of September 13, 2018 data cut), 3 partial responses were observed (34%). 4/9 (45%) pts had stable disease (SD), 2 of SD patients are experiencing tumor reduction (>20%). No Grade ≥3 treatment-related adverse events (TRAEs) were reported.

**Conclusions**

APX005M in combination with pembrolizumab is well tolerated and has clinical activity. Updated safety, biomarker and response data will be presented.

**Trial Registration**

NCT: 02706353

## P711 HepaVac-101 first-in-man therapeutic cancer vaccine Phase I/II clinical trial for hepatocellular carcinoma patients

### Luigi Buonaguro, MD^1^, Andrea Mayer-Mokler^2^, Roberto Accolla^2^, Yuk T. Ma^4^, Regina Heidenreich^5^, Antonio Avallone^1^, Ester Simeone^1^, Alfred Koenigsrainer^6^, Markus Loeffler^6^, Cecile Gouttefangeas, PhD^6^, Christian Flohr^2^, Jörg Ludwig^2^, Diego D. Alcoba, MSc, PhD^2^, Sarah Kutscher^2^, Maria Tagliamonte^1^, Paolo A. Ascierto^1^, Hans-Georg Rammensee, PhD^6^, Bruno Sangro^7^, Mercedes Iñarrairaegui Bastarrica^7^, Sven Francque, MD, PhD^8^, Luisa Vonghia^8^, Danila Valmori^9^, Tanguy Chaumette^9^, Toni Weinschenk, PhD^2^, Carsten Reinhardt, MD, PhD^2^, Ulrike Gnad-Vogt, MD^5^, Luigi Buonaguro, MD^1^, Harpreet Singh-Jasuja^2^

#### ^1^National Cancer Institute ‘Pascale’, Napoli, Italy; ^2^IMMATICS Biotechnologies GmbH, Tuebingen, Germany; ^2^University of Insubria, Varese, Italy; ^4^University of Birmingham, Birmingham, UK; ^5^CUREVAC AG, Tuebingen, Germany; ^6^Univ. Hospital Tuebingen, Tuebingen, Germany; ^7^University of Navarra, Pamplona, Spain; ^8^Antwerp Univ. Hospital, Antwerp, Belgium; ^9^University of Nantes, Nantes, France

##### **Correspondence:** Luigi Buonaguro (l.buonaguro@istitutotumori.na.it)

**Background**

Hepatocellular carcinoma (HCC) is the third leading cause of death from cancer globally with an extremely variable 5-year survival rate. Immunotherapy strategies for HCC may represent a key therapeutic tool to improve clinical outcome in HCC patients. The HepaVac-101 phase I/II, first-in-man, single-arm clinical trial is performed as part of the HepaVac project, funded by the European Commission’s 7th Framework Program under the Grant Agreement Nr. 602893 (www.hepavac.eu). The HepaVac-101 trial identification numbers are NCT03203005 (Clinical trials.gov) and 2015-003389-10 (EudraCT).

**Method**

The therapeutic cancer vaccine IMA970A is a multi-peptide-based HCC vaccine composed of 16 newly discovered and overexpressed tumor-associated peptides (TUMAPs) directly identified from resected HCC tissues. Of these TUMAPs, 7 are restricted to HLA-A*02, 5 to HLA-A*24 and 4 to HLA class II. CV8102 is a novel ribonucleic acid (RNA) based immunostimulatory agent inducing a balanced Th1/Th2 immune response. Patients with very early, early and intermediate stage of HCC are enrolled to be treated with a single pre-vaccination infusion of low-dose cyclophosphamide, followed by 9 intradermal vaccinations consisting of IMA970A plus CV8102. The study drugs are applied without concomitant anti-tumor therapy, in order to reduce risk of tumor recurrence/progression in patients having received all indicated standard treatments and without evidence of active disease. The primary endpoints of the HepaVac-101 clinical trial are safety, tolerability, and immunogenicity. Secondary/exploratory endpoints are additional immunological parameters in circulation (e.g. regulatory T-cells, myeloid-derived suppressor cells, impact of the standard therapy on the natural immune response), infiltrating T-lymphocytes in tumor tissue, biomarkers in blood and tissue, disease-free survival/progression-free survival and overall survival. Overall, it is planned to enroll about 20 to 40 patients. Suitable patients enrolled at Tuebingen are invited to participate in a trial extension investigating an actively personalized vaccine (APVAC). The HepaVac-101 trial is conducted in 6 centers located in 5 European countries. Five centers are actively recruiting patients and one additional site will start enrollment in Q3 2018. As of the time of abstract submission, 42 HCC patients have been screened for HLA haplotype. Two patients are engaged in the vaccination protocol and one patient has completed the study treatment (currently on follow-up phase).

## P712 Early phase 2 clinical results of IL-15RαFc superagonist N-803 with BCG in BCG-unresponsive non-muscle invasive bladder cancer (NMIBC) patients demonstrating 86% CR of carcinoma in situ (CIS)

### John Lee, MD^1^, Patrick Soon-Shiong, MD^1^, FRCS, FACS, Karim Chamie, MD^2^, Amy Rock, PhD^1^, Peter Rhode, PhD^3^

#### ^1^NANT Cancer Immunotherapy Inc., Culver City, CA, USA; ^2^Ronal Reagan UCLA Medical Center, Los Angeles, CA, USA; ^3^NantCell, Culver City, CA, USA

##### **Correspondence:** John Lee (John.Lee@NantKwest.com)

**Background**

Patients with BCG-unresponsive non-muscle-invasive bladder cancer (NMIBC) have limited treatment options and the standard of care is radical cystectomy. N-803 is an IL-15-based immunostimulatory protein complex (IL-15RαFc) that promotes proliferation and activation of natural killer (NK) cells, effector and memory CD8+ T cells, but not Treg cells. Preclinical data have shown that when combined with BCG, N-803 activates natural killer cells and reduces tumor burden [1]. Phase Ib data in BCG-naïve patients with NMIBC demonstrate that intravesical administration of N-803 with BCG induced complete response in all patients, without recurrences for more than 24 months [2]. A patient with high-risk NMIBC who had failed multiple intravesical therapies remained disease-free for over 19 months when treated with N-803 and BCG [3].

**Method**

Based on these studies fast track designation was obtained and an open label single arm multicenter Phase 2 study of intravesical BCG plus N-803 in patients with BCG unresponsive high grade NMIBC (NCT03022825) was opened. Two study groups: group A patients with BCG-unresponsive carcinoma in situ (CIS) [with or without Ta or T1 disease] and group B patients with BCG-unresponsive high-grade Ta or T1 disease [no CIS]. All patients treated received intravesical N-803 plus BCG, weekly for 6 consecutive weeks during the induction treatment period. First response assessment at Week 12. Patients with no disease or low-grade Ta disease at months 6, 9, 12, and 18 are eligible for continued maintenance treatment. The primary endpoint is incidence of complete response of CIS at any time.

**Results**

To date, twenty-two patients have enrolled in the phase 2 trial (Group A (CIS), n=11, Group B (Papillary), n=11). Seven of the eleven patients in Group A with BCG-unresponsive CIS have reached at least the 12 week response assessment timepoint. Of these seven patients, six patients (86%) have a reported complete response. Eight of the eleven patients with BCG-unresponsive high-grade Ta or T1 disease have evaluable data. None of the eight have experienced recurrence of disease. Two serious adverse events (AEs) have been reported (E coli infection, anemia), with no immune related AEs. In Group B, not a single patient out of eleven has recurred on study to date (3-12 months since resection).

**Conclusions**

Early objective response in patients with NMIBC unresponsive to BCG with CIS demonstrated a complete response in 6 out of the first 7 patients (86%). Furthermore, these patients demonstrated no immune related AEs. In patients with papillary, no recurrences to date.

**Trial Registration**

NCT03022825

**References**

1. Gomes-Giacoia E, Miyake M, Goodison S, Sriharan A, Zhang G, You L, Egan JO, Rhode PR, Parker AS, Chai KX, Wong HC, Rosser CJ. Intravesical ALT-803 and BCG treatment reduces tumor burden in a carcinogen induced bladder cancer rat model, a role for cytokine production and NK cell expansion. PLoS One. 2014 Jun 4,9(6):e96705.

2. MP-15-12: Phase 1b trial of ALT-803, an IL-15 superagonist, plus BCG for the treatment of BCG-naïve patients with non-muscle invasive bladder cancer. Rosser CJ, Nix J, Ferguson L, Wong HC, The Journal of Urology, Vol. 197, Issue 4, e175 Published in issue: April 2017.

3. Huang J, Schisler J, Wong HC, Rosser CJ, Sterbis J. Intravesical ALT-803 for BCG-unresponsive Bladder Cancer - A Case Report. Urol Case Rep. 2017 Jun 8,14:15-17.

## P713 NANT Cancer Vaccine an orchestration of immunogenic cell death by overcoming immune suppression and activating NK and T cell therapy in patients with third line or greater metastatic pancreatic cancer

### Patrick Soon-Shiong, MD, FRCS, FACS^1^, John Lee, MD^1^, Tara Seery, MD^2^, Mira Kistler, MD^2^, Arvind Shinde^2^, Anand Annamalai^2^, Leonard Sender^2^, Frank Jones, PhD^3^, Omid Jafari, MD^4^

#### ^1^Nantkwest, Culver City, CA, USA; ^2^Chan Soon-Shiong Institute for Medicine, el Segundo, CA, USA; ^3^NantCell, Culver City, CA, USA; ^4^Medical Imaging Center of SoCal, Los Angeles, CA, USA

##### **Correspondence:** Patrick Soon-Shiong (pss@nantworks.com)

**Background**

Pancreatic cancer has multiple mechanisms to prevent immune recognition that lead to the creation of an immune suppressive tumor microenvironment. We hypothesize that effective and sustained response against tumors requires a coordinated approach that: 1. reverses the immune-suppressive tumor microenvironment, 2. induces immunogenic tumor cell death and 3. reengages NK and T-cell tumor response against a 4. cascade of tumor antigens. To test this hypothesis, we have developed the NANT Cancer Vaccine: a temporospatial approach that combines: metronomic low dose chemotherapy, SBRT, off-the-shelf cryopreserved allogeneic NK cells, yeast and adenoviral tumor associated antigen vaccines, IL-15RαFc superagonist N-803 immunostimulatory cytokine, with checkpoint inhibitor.

**Method**

A phase 1b, single-arm, open-label trial of the NANT Cancer Vaccine in patients with recurrent metastatic pancreatic cancer was initiated. Treatment occurred in 3-week cycles of low-dose chemotherapy (aldoxorubicin, cyclophosphamide, oxaliplatin, nab-paclitaxel, 5-FU/L), antiangiogenic therapy (bevacizumab), SBRT, engineered allogeneic high affinity CD-16 NK-92 cells (haNK), IL-15RαFc (N-803), adenoviral vector-based CEA vaccine (Ad-CEA), yeast vector-based RAS vaccine (Ye-RAS), and an IgG1 PD-L1 inhibitor, avelumab. The primary endpoint is incidence of treatment-related adverse events. Secondary endpoints include ORR, DCR, PFS, and OS.

**Results**

To date, 10 patients with 3rd-line or greater metastatic pancreatic cancer have initiated treatment with response evaluated past 10 weeks. All therapies were safely administered in an outpatient setting. AEs were primarily hematologic which were managed by appropriate planned dose chemo reduction. No dose-limiting, immune-related AE’s (irAE’s) have been observed to date. Eight out of ten patients (80%) have had a best response of stable disease with a 100% disease control of target lesions to date. Median progression-free survival is 5.8 months (3.3 – 8.8) and median overall survival is 9.5 months (5.0 – NR) with patients continuing treatment.

**Conclusions**

This preliminary data suggests that the NANT Cancer Vaccine of low-dose chemo-radiation combined with innate and adaptive immunotherapy can be administered safely in an outpatient setting. Preliminary efficacy results are encouraging and the overall survival of 9.5 months currently exceeds all standards of care for patients at this advanced stage of disease.

**Trial Registration**

NCT03387098

## P714 Phase 2 trial of CA-170, a novel oral small molecule dual inhibitor of immune checkpoints VISTA and PD-1, in patients (pts) with advanced solid tumor and Hodgkin lymphoma.

### Vivek S. Radhakrishnan, MD, DM^1^, Sameer Bakhshi, MD^2^, Kumar Prabhash, MD^3^, Chetan Deshmukh, MD^4^, Shona Nag, MD^5^, KC Lakshmaiah, MD^6^, M Gopichand, MCh^7^, Murali Ramachandra, PhD^8^, Sudeep Gupta, MD^9^, Shripad D. Banavali, MD^3^, Divyesh Mandavia^8^, Akhil Kumar, MD^10^

#### ^1^TMC Kolkata, Kolkata, India; ^2^AIIMS, New Delhi, India; ^3^TMH Mumbai, Mumbai, India; ^4^Deenanath Mangeshkar Hospital, Pune, India; ^5^Jehangir Hospital, Pune, India; ^6^Sreenivasam Cancer Care Hospitals, Bengaluru, India; ^7^City Cancer Centre, Vijayawada, India; ^8^Aurigene Discover Technologies Limited, Bangalore, India; ^9^ACTREC, Navi Mumbia, India; ^10^Advisor-Aurigene, Bengaluru, India

##### **Correspondence:** Murali Ramachandra (murali_r@aurigene.com)

**Background**

V-domain Ig suppressor of T-cell activation (VISTA) and Programmed-death 1 (PD-1) are independent immune checkpoints that negatively regulate T-cell function and are implicated in various malignancies. Preclinical studies have demonstrated that dual blockade of these pathways is synergistic. CA-170 is a first-in-class oral small molecule that directly targets both VISTA and PD-1/PD-L1 pathways and has shown anti-tumor activity in multiple preclinical models. A Phase 1 dose escalation study (Clinicaltrials.gov NCT02812875) has shown acceptable safety of CA-170 with dose escalated up to 2400mg daily.

**Method**

The Phase II study is a multi-tumor (Head & Neck Cancer, Squamous-NSCLC, Non-Squamous-NSCLC, MSI-H positive solid tumors and Hodgkin Lymphoma) Simon Two Stage design, investigating two dosages (400mg versus 800mg) of CA-170. The total sample size, if all tumor types go into Simon Stage 2, is 130. Key eligibility criteria include: age ≥ 18 years, ECOG ≤1, adequate organ function, no previous exposure to immuno-oncology agents, and 1-3 prior lines of systemic therapy. Primary objective is efficacy, as measured by response rates. Secondary endpoints include additional efficacy measures, as well as safety and PK/PD endpoints.

**Results**

As of September 13, 2018, 58 patients (18 Head & Neck Cancer, 5 Squamous-NSCLC, 18 Non-Squamous NSCLC, 5 MSI-H positive Solid Tumors and 12 Hodgkin Lymphoma) have been enrolled. Out of these 22 patients have had at least one follow up scan. Among the patients who have had follow up scans, significant anti-tumor activity is seen in three patients – two Hodgkin lymphoma patients showing partial response by Lugano criterion and a Head and Neck Cancer patient showing 48.1% reduction in SPD by irRC criterion. The Clinical Benefit Rate (SD or better) is 68.18%. The AEs and SAEs have been as expected in this population, without any concerns. With respect to immune related adverse events, two patients developed skin rash, one patient developed hypothyroidism, and one patient developed Grade 3 neutropenia and anemia. Both anemia and neutropenia resolved upon drug interruption, however, neutropenia re-appeared after re-exposure, confirming causality. CA-170 was permanently discontinued without any sequalae, suggesting that smaller half life (6-8 hours) of CA-170 provides an advantage over longer lasting antibodies, from safety perspective.

**Conclusions**

To our knowledge, this is the first Phase 2 study of an oral immuno-oncology (IO) agent, showing activity in cancer patients. Updated efficacy and safety data will be presented at the meeting.

**Acknowledgements**

All the patients and institutes which participated in the study

**Ethics Approval**

The study was approved by the respective Ethics Committee of each of the 15 participating institutions.

**Trial Registration**

Clinical Trials Registry - India (CTRI/2017/12/011026)

## P715 A Phase II study of bemcentinib (BGB324), a first-in-class selective AXL inhibitor, in combination with pembrolizumab in patients with advanced NSCLC: Analysis of the first stage

### Matthew G. Krebs, MD PhD^1^, Paal F. Brunsvig, MD, PhD^2^, Nuria Vinolas Segarra^3^, Luis Paz-ares^4^, Enric Carcereny^5^, Enriqueta Felip, MD PhD^6^, Manuel Dómine Gómez, MD PhD^7^, José Trigo, MD^8^, Edume Arriola, MD PhD^9^, Maria Rosario Garcia Campelo, MD PhD^10^, James Spicer, MD, PhD^11^, Jonathan Thompson, MD MS^12^, Konstantin Dragnev, MD^13^, David Micklem, PhD^14^, Robert Holt, PhD^14^, Anthony Brown^14^, James Lorens, PhD^15^, Michael J. Chisamore, PhD^16^, Matthew G. Krebs, MD PhD^1^

#### ^1^The Christie NHS Foundation Trust, Manchester, UK; ^2^Oslo University University Hospital, Oslo, Norway; ^3^Hospital Clinic de Barcelona, Barcelona, Spain; ^4^ Hospital Universitario Virgen del Rocio, Sevilla, Spain; ^5^Hospital Universitari Germans Trias, Barcelona, Spain; ^6^Hospital Unviersitario Val d’Hebron, Barcelona, Spain; ^7^ Hospital Unviersitario Fundacion Jimenez, Madrid, Spain; ^8^Hospital Universitartio Virgen de la Victoria, Malaga, Spain; ^9^Hospital del Mar, Barcelona, Spain; ^10^Hospital Teres Herrera/CHUAC, A Coruña, Spain; ^11^Guy’s and St Thomas’ NHS Foundation, London, UK; ^12^Medical College of Wisconsin, Milwaukee, WI; ^13^Dartmouth-Hitchcock Medical Center, Hanover, NH, USA; ^14^BerGenBio ASA, Bergen, Norway; ^15^University of Bergen, Bergen, Norway; ^16^Merck & Co, Inc., Rahway, NJ, USA

##### **Correspondence:** James Lorens (james.lorens@uib.no)

**Background**

AXL kinase suppresses innate immune response and its over-expression has been observed in patients failing anti-PD-1 therapy. Bemcentinib (BGB324) is a first-in-class, oral, highly selective AXL inhibitor in phase II clinical development. We evaluated the safety and preliminary efficacy of bemcentinib in combination with the anti-PD-1 pembrolizumab in NSCLC patients.

**Method**

Patients with stage IV lung adenocarcinoma, unselected for PD-L1, who progressed following 1L platinum doublet chemotherapy were eligible. Bemcentinib (200mg) was administered daily with 3-weekly pembrolizumab (200mg) until disease progression, unacceptable toxicity, or withdrawal.The primary endpoint was ORR (RECIST v1.1), additional objectives included DCR, PFS, OS and safety of the combination.A two-stage Simon-like design was employed (48 patients for a H0 of RR≤0.15, p=0.1, 80% power) with an interim futility analysis after the first 22 patients had had the potential for 24 weeks of follow-up.Fresh pre-treatment tumour biopsies were mandatory for PD-L1 (22C3 pharmDx assay, Agilent) and AXL analysis by IHC (Indivumed).

**Results**

As of 3 Sep 2018, enrolment into the first stage was complete. Twenty-four patients were recruited (PD-L1 negative: 11 (46%), weak positive (TPS: 1 - 49%): 7 (29%), strong positive (TPS: >50%): 2 (8%)) and had completed an average of 4.2 cycles of treatment (range 0 – ongoing), five patients remained on treatment.In the total (intention-to-treat) population, ORR was 21% (5/24) with DCR of 54% (PR+SD = 13/24) (Table 1). Median PFS was 4.0 months (95% CI 2.0 – NR). Among 21 patients evaluable for AXL status, 10 had AXL positive tumour tissue by IHC. Efficacy was higher in AXL positive patients with ORR 40% (4/10) and DCR 70% (7/10). Of note, 4 (40%) of these patients were PD-L1 negative. This compared with an ORR of 9% (1/11) and DCR 45% (5/11) in AXL negative patients of whom 6 (55%) were PD-L1 negative. Median PFS was 5.9 months (1.7 – NR) in AXL positive patients versus 3.3 months (1.2 - NR) in AXL negative. Treatment was generally well tolerated with no grade 4 or 5 treatment related adverse events (TRAEs). Most common TRAEs were diarrhea (25%) and elevated alanine aminotransferase (25%).

**Conclusions**

Promising clinical activity was seen particularly in patients with AXL positive disease including those with weak or no PD-L1 expression. The combination treatment of bemcentinib and pembrolizumab was overall well tolerated. Efficacy, safety and putative biomarker data will be further explored in stage 2 of the study.

**Acknowledgements**

The authors would like to thank all patients and their caretakers for participating in this trial.

**Ethics Approval**

All relevant approvals have been obtained.

**Trial Registration**

NCT03184571

**Table 1 (abstract P715).**
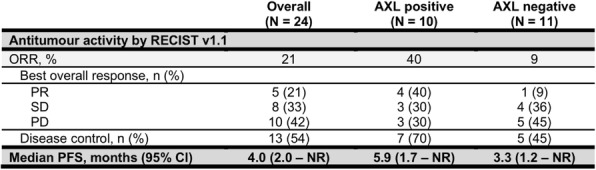
Stage 1 Efficacy Results Overall and by AXL Status

## P716 The first clinical/translational data from the expansion cohorts of a Ph1/1b Study of IPI-549, a tumor macrophage-reprogramming small molecule, in combination with nivolumab in advanced solid tumors

### Bartosz Chmielowski, MD, PhD^1^, Ryan J. Sullivan, MD^2^, Michael Postow, MD^3^, Amita Patnaik, MD FRCP(C)^4^, Geoffrey Shapiro, MD, PhD^5^, Ezra Cohen, MD^6^, Martin Gutierrez, MD^7^, Conor Steuer, MD^8^, Antoni Ribas, MD, PhD^1^, Lucy Lee, PHARMD, DABCP, FCP^9^, Brenda O'Connell, PhD^9^, Jeffery Kutok, MD, PhD^9^, Jennifer Roberts^9^, Suresh Mahabhashyam, MBBS^9^, Marie-Louise Fjallskog, MD^9^, Jedd D. Wolchok, MD, PhD^3^, David Hong, MD^10^

#### ^1^University of California, Los Angeles, CA, USA; ^2^Massachusetts General Hospital, Boston, MA, USA; ^3^Memorial Sloan Kettering Cancer Center, New York, NY, USA; ^4^South Texas Accelerated Research Therapeutics (START), San Antonio, TX, USA; ^5^Dana-Farber Cancer Institute, Boston, MA, USA; ^6^University of California, San Diego, San Diego, CA, USA; ^7^Hackensack University Medical Center, Hackensack, NJ, USA; ^8^Emory University School of Medicine, Atlanta, GA, USA; ^9^Infinity Pharmaceuticals, Cambridge, MA, USA; ^10^MD Anderson Cancer Center, Houston, TX, USA

##### **Correspondence:** Jennifer Roberts (jennifer.roberts@infi.com)

**Background**

IPI-549 is a first-in-class, oral, selective PI3K-γ inhibitor. In preclinical studies IPI-549 reprograms macrophages/myeloid derived suppressor cells (MDSCs) from an immune-suppressive to an immune-activating phenotype and overcomes resistance to checkpoint inhibitors.

**Method**

Study IPI-549-01 (NCT02637531) is evaluating the safety, tolerability, pharmacokinetics/pharmacodynamic, immunomodulatory activity and efficacy of IPI-549 in advanced solid tumor patients, as monotherapy and in combination with nivolumab. The recommended dose for expansion was IPI-549 40 mg QD PO + nivolumab 240 mg Q2W IV. The trial is currently enrolling 6 disease-specific combination expansion cohorts including patients with intrinsic and acquired resistance to anti-PD(L)1 therapy, and a 7th combination expansion cohort with patients prospectively selected for high blood MDSC levels (>20.5%). Pre- and on-treatment blood samples for flow cytometry, gene expression, and serum analysis, and paired biopsies for gene expression and immunohistology are mandated.

**Results**

As of 22 Aug 2018, 57 patients have been enrolled in the combination expansion cohorts: triple-negative breast cancer (TNBC) (n=13), mesothelioma (11), melanoma (11), head and neck squamous cell carcinoma (9), high MDSCs (7), adrenocortical carcinoma (4), and non-small cell lung cancer (2). Clinical activity data is available in 27 evaluable ( >1 scan) patients and include two PRs: 1/7 patients in the anti-PD(L)-1 therapy refractory melanoma cohort and 1/6 patients in the anti-PD(L)1 therapy naïve TNBC cohort. Most adverse events (AEs) were Grade 1-2 in severity. The most common treatment-related adverse events (TRAEs) included rash (24%), fatigue (17%), AST increased (17%), ALT increased (14%), pyrexia (14%), and ALP increased (12%), and the most common Grade 3-4 TRAEs included rash (11%) and increased AST (9%), ALP (7%) and ALT (5%). There were no treatment-related deaths.Translational data is being evaluated for alterations in MDSCs, interferon-gamma-induced proteins and proliferation of previously exhausted CD8 memory T-cells in blood, as well as changes in immune infiltrates from paired tumor biopsies Currently, translational data is available in the anti-PD1 therapy refractory melanoma patient showing an 86% reduction in MDSCs at C2D1 by flow cytometry and a 68% increase in the proliferative fraction of previously exhausted T-cells at C3D1 relative to baseline.Updated clinical and translational data from the combination expansion cohorts will be presented.

**Conclusions**

The combination of IPI-549 + nivolumab demonstrates an acceptable safety profile and clinical activity associated with on-mechanism immunomodulation in patients not expected to respond to PD1 inhibition alone, including a PR in a melanoma patient progressing on immediate prior nivolumab.

## P717 Initial report of intratumoral tavokinogene telseplasmid with pembrolizumab in advanced melanoma: an approach designed to convert PD-1 antibody progressors into responders. (NCT 03132675)

### Sharron E. Gargosky, PhD^1^, Victoria Atkinson, MD^2^, Andrew Haydon, MBBS PhD^3^, Phillip Parente^4^, Tom Van Hagen^5^, Gregory A. Daniels, MD, PhD^6^, Pablo Fernandez-Penas, MD, PhD^7^, Mecker Moller^8^, Igor Puzanov, MD, MSCI, FACP^9^, Sajeve Thomas^10^, Robert H. Andtbacka, MD, CM, FACS, FRCSC^11^, Clemens Krepler, MD^13^, David A. Canton, PhD^1^, Christopher Twitty, PhD^1^, Sharron E. Gargosky, PhD^1^

#### ^1^Oncosec Medical Incorporated, San Diego, CA, USA; ^2^Princess Alexandra Hospital, Woolloongabba, Australia; ^3^The Alfred Hospital, Melbourne, Australia; ^4^Eastern Health Monash University, Box Hill, Australia; ^5^4St. John of God Hospital, Subiaco, Australia; ^6^University of California San Diego, San Diego, CA, USA; ^7^Westmead Hospital, Westmead, Australia; ^8^University of Miami, Miami, FL, USA; ^9^Roswell Park Comprehensive Cancer Center, Buffalo, NY, USA; ^10^UF Health Cancer Center at Orlando Health, Orlando, FL, USA; ^11^University of Utah, Salt Lake City, UT, USA; ^13^Merck & Co. Inc., Kenilworth, NJ, USA

##### **Correspondence:** Christopher Twitty (chrisgtwitty@gmail.com)

**Background**

Anti-PD-1 antibodies are the mainstay of treatment for advanced melanoma, but PD-1 resistance has emerged as a major mechanism that limits clinical benefit in a majority of treated patients. Absence of T cell-inflamed lesions have emerged as a key determinant of this poor response. Delivery of tavokinogene telseplasmid (tavo) intratumorally by electroporation (IT-tavo-EP) produces expression of the proinflammatory cytokine IL-12, which can convert treated and non-treated, weakly immunogenic/poorly T cell-inflamed tumors into highly inflamed, immunologically active lesions. The current trial examines whether IT-tavo-EP can reverse resistance to anti-PD-1 therapies (NCT 01313267).

**Method**

PISCES is a multicenter phase 2, open-label trial of IT-tavo-EP with pembrolizumab in patients with stage III/IV melanoma who have progressed on either pembrolizumab or nivolumab mono- or combination therapy. The primary endpoint is ORR by RECIST v1.1 at 24 weeks as determined by central review. 48 eligible patients will be treated with IT-tavo-EP to accessible lesions on Days 1, 5 and 8 every 6 weeks combined with IV pembrolizumab (200 mg) on Day 1 of each 3-week cycle for 24 weeks. Longitudinal blood, tissue and fecal samples are being collected and will be used to explore immunological mechanism via transcriptional analyses, multispectral IHC, immune phenotypic analysis, and TCRβ sequencing.

**Results**

The current data comprises 21 patients with the first 9 patients having completed 12 weeks of treatment. Of the 21 patients, the average age is 63 +/- 12 years with 62% (13/21) males. All patients have previously been treated and progressed on anti-PD-1 therapies with 36% (7/19) having had more than one prior line of therapy. Immunologically, all enrolled patients had exceedingly low frequencies of intratumoral peCTL (PD-1+/CTLA-4+CD8+ T cells) at screening with a notable increase in TIL density with treatment. Tavo dosing ranged 0.1-12.5mL per lesion. AEs were predominantly grade 1 associated with injection site or procedural pain. One expected SAE of cellulitis (grade 3) has been reported and two unrelated SAEs (grade3) of respiratory tract infection and elective surgery, all resolved. Of the first 9 patients who had completed 12 weeks of treatment, Tumor responses (RECIST v1.1) were noted in both treated and non-treated lesions with 2 partial responses and 1 stable disease being observed per investigator assessment. These responses were associated with treatment-related upregulation of immune-based transcripts in the tumor microenvironment as well as increased intratumoral T cells within 3 weeks of therapy.

**Conclusions**

The trial is showing early signs of reversing resistance and continues enrolling.

**Ethics Approval**

WIRB protocol #20171064

## P718 Profiling PD-1 blockade pharmacodynamics using a live tissue explant model of head and neck squamous cell carcinoma

### Munisha Smalley^1^, PharmD, Steven B. Maron, MD^2^, Manjusha Biswas, MD^1^, Mark Lawson, BA, HTL(ACSP) ^1^, Biswanath Majumder^1^, Basavaraja Uddajjara Shanthappa^1^, Saravanan Thiyagaragan^1^, Kodaganur S. Gopinath^3^, K.S. Sabitha^4^, Joseph F. Grosso, PhD^5^, Tanguy Seiwert, MD^2^, Aaron Goldman^6^

#### ^1^Mitra Biotech, Woburn, MA, USA; ^2^University of Chicago, Chicago, IL, USA; ^3^HCG Oncology Centre, Bangalore, India; ^4^Kidwai Memorial Insitute of Oncology, Bangalore, India; ^5^Bristol Myers Squibb, Lawrenceville, NJ, USA; ^6^Brigham and Women’s Hospital, Boston, MA, USA

##### **Correspondence:** Aaron Goldman (goldman1@mit.edu)

**Background**

Immune checkpoint inhibitors revolutionized cancer immunotherapy, yet clinical success remains highly variable and often patient-specific. Therefore, a method to study the mode-of-action of checkpoint blockade at the individual patient level is a critical step towards effectively personalizing immuno-oncology.

**Method**

Here, we characterized the immune fidelity of CANscript, a live tissue explant model that harnesses a clinically-trained algorithm to predict tumor response to therapy (a.k.a M-Score). We profiled spatio-temporal immune cell heterogeneity using multi-spectral imaging and gene expression analysis. In addition, we examined how well functional tumor-immune biology was preserved across the lymphoid and myeloid compartments during ex-vivo culture using multiple different platforms including cytokine multiplex analysis, flow cytometry, gene expression quantification and multispectral immunohistochemistry. In this platform, we interrogated clinically-approved PD-1 inhibitors (e.g. Nivolumab and Pembrolizumab) using tumor biopsies from patients (N=50) with head and neck squamous cell carcinoma (HNSCC). To buttress these data, and provide a clinical translation, we profiled 12 patient samples in the explant model in which we also obtained the matched clinical response.

**Results**

We show that in vivo spatial T-cell heterogeneity, including CD4 and CD8 T-cell distributions, tumor-immune pathways and lymphocyte lineage differentiation were recapitulated by CANscript. We identified robust retention of the tumor-immune contexture during the ex-vivo tumor culture (72 hours), and found a statistically significant correlation between the baseline heterogeneity of the tumor microenvironment (when the tissue arrived at the lab) across gene and protein expression as well as preservation of immune cell signaling networks. Furthermore, we determined that spatial heterogeneity of immune cells within the tumor was unique to each patient, which was also retained after culture. Following treatment with PD-1 inhibitors, we identified high inter-patient gene and protein expression variability is induced by drug pressure, which was predominated by a shift in T-helper and activated T-cell activity particularly Th1 and Th2. Using a clinically-trained algorithm that predicts clinical response, we stratified samples further to study pharmacodynamics of PD-1 blockade, identifying a subset of patient samples that induce adaptive immune response and inflammatory cytokines, which associated to drug efficacy vs. resistance. Induction of T-helper cell gene signatures, particularly a Th1-Hi phenotype from the ex-vivo model were validated using tumor samples from patients ON-TREATMENT.

**Conclusions**

These findings highlight the now-obvious need to profile efficacy of immunotherapy at the individual patient level, and the utility of ex-vivo tumor models with high immune-fidelity to advance the goals of personalized immuno-oncology.

**Ethics Approval**

The study was approved by IRB at Kidwai and HCG Oncology Hospitals, Bangalore India.

## P719 A SITC-sponsored randomized clinical trial to determine criteria to guide clinicians on when to stop immunotherapy through a community-driven data repository, leveraging the SITC community

### Jennifer L. Guerriero, PhD^1^, Jessica Thaxton, PhD, MSCR^2^, Todd Bartkowiak^3^, Esha Sachdev, MD^4^, Jiajia Zhang, MD, MPH^5^, Abdul Rafeh Naqash, MD^6^, Rania H. Younis, BDS, MDS, PhD^7^, Sarah E. Church^8^, Maria E. Rodriguez-Ruiz, MD, PhD^9^, Rosa Nguyen^10^, Kit Fuhrman, PhD^8^, Sabrina Kaczanowska^11^, Abigail E. Overacre-Delgoffe, PhD^12^, Dipti Thakkar, PhD^13^, Yinghong Wang, MD, PhD^14^, Aideen E. Ryan, PhD^15^, Claire A. Margolis, MS^1^, Rachel Howard^16^, Daniel J. Olson, MD^17^, Michal Sheffer, PhD^1^, Kristin G. Anderson, PhD^18^, Yuanquan Yang, MD, PhD^19^, Namrata S. Chandhok^20^, Vaia Florou, MD^21^, Sangeetha M. Reddy, MD, MSci^14^, David H. Aggen, MD, PhD^22^, Ravi Patel, MD, PhD^23^, Thomas U. Marron, MD PhD^24^

#### ^1^Dana-Farber Cancer Institute, Boston, MA, USA; ^2^Medical University of South Carolina, Columbia, SC, USA; ^3^Vanderbilt University, Nashville, TN, USA; ^4^University of California, Los Angeles, Los Angeles, CA, USA; ^5^John Hopkins School of Medicine, Baltimore, MD, USA; ^6^East Carolina University, Greenville, NC, USA; ^7^University of Maryland Baltimore, Baltimore, MD, USA; ^8^Nanostring Technologies, Inc., Seattle, WA, USA; ^9^Clinica Universidad de Navarra, Pamplona, Spain; ^10^St. Judes’ Children’s Research Hospital, Memphis, TN, USA; ^11^National Cancer Institute, Bethesda, MD, USA; ^12^Children’s Hospital of Pittsburgh, Pittsburgh, PA, USA; ^13^Hummingbird Bioscience, Singapore, Singapore; ^14^MD Anderson Cancer Center, Houston, TX, USA; ^15^NUI Galway, Galway, Ireland; ^16^H. Lee Moffitt Cancer Center & Research, Tampa, FL, USA; ^17^Univeristy of Chicago, Chicago, IL, USA; ^18^Fred Hutchinson Cancer Research Center, Seattle, WA, USA; ^19^Roswell Park Cancer Institute, Buffalo, NY, USA; ^20^Yale Univeristy, New Haven, CT, USA; ^21^University of Miami, Miami, FL, USA; ^22^Columbia Univeristy Medical Center, New York, NY, USA; ^23^University of Wisconsin – Madison, Madison, WI, USA; ^24^Icahn School of Medicine at Mount Sinai, New York, NY, USA

##### **Correspondence:** Jennifer L. Guerriero (Jennifer_Guerriero@dfci.harvard.edu)

**Background**

SITCure: Sparkathon 2018: When is it safe to stop immunotherapy?

Metastatic melanoma patients have received immense clinical benefit from checkpoint blockade therapies that have extended long-term survival rates. This advance in cancer treatment has left clinicians and patients in uncharted territory, questioning when it is safe to stop treatment. While many patients achieve maximal response within 6-8 months from the start of treatment, most clinical trials have treated patients for 24 months or indefinitely until progression. Continued treatment beyond best response may provide little additional clinical benefit, result in late-onset immune-related adverse events, and pose excessive personal and societal financial burden. While some physicians opt to stop therapy at or before 24 months of treatment, there are currently no available data-driven guidelines to inform these decisions, therefore, a standardized guideline is critical.

**Method**

To improve quality of care for patients and financial burden on the health care system, we have designed a clinical trial to guide when to stop immunotherapy through a community driven data resource, leveraging the SITC community.

Clinical trial design: This study will challenge the standard-of-care for anti-PD-1 treatment with a non-inferiority multi-institutional trial. The trial will enroll patients with metastatic melanoma that have received 1-year of standard anti-PD-1 therapy and have stable disease for at least 3 months—having achieved complete response, partial response, or stable disease. Patients will be randomized to a 2-year observational arm or will continue anti-PD-1 for one additional year, after which therapy will be discontinued. Retrospective medical history and imaging will be obtained, and prospective blood, serum, and stool will be collected every 3 months (Figure 1). The primary endpoint will be time to progression following randomization. Secondary endpoints will be 3-year overall and progression-free survival.

Data Analysis: Steering Committees spearheaded by SITC Sparkathon Class of 2018 will be formed and will drive development of standard operating procedures for collection, processing and storage. The Steering Committees encompassing a broad range of scientific expertise (Figure 2), will determine metrics used to analyze specimens, identify biomarkers of remission or risk of recurrence upon discontinuation of anti-PD-1, and test hypotheses to determine safety guidelines for termination of therapy. All data obtained from sample analysis will be deposited into an open-access data repository available to the larger SITC community. Steering Committees will design and test hypotheses to identify discrete markers or signatures that determines when/if it is safe for clinicians to discontinue immunotherapy.

**Results**

N/A

**Conclusions**

N/A

**Acknowledgements**

We would like to thank our business leader, David A. Rosen, for his time and patience in helping us create this proposal and the SITC Sparkathon staff for their support.

**Fig. 1 (abstract P719).**
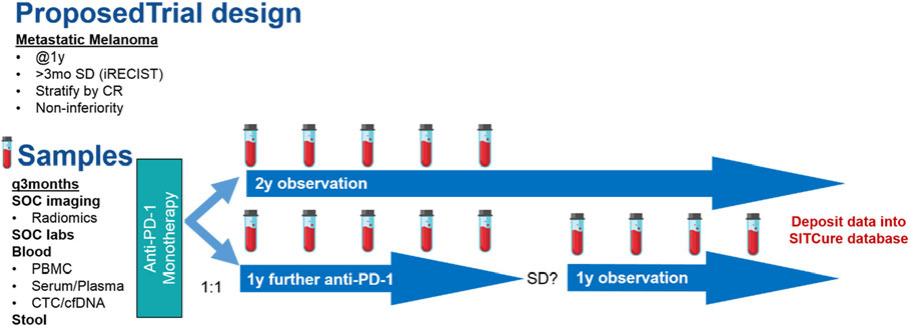
See text for description

**Fig. 2 (abstract P719).**
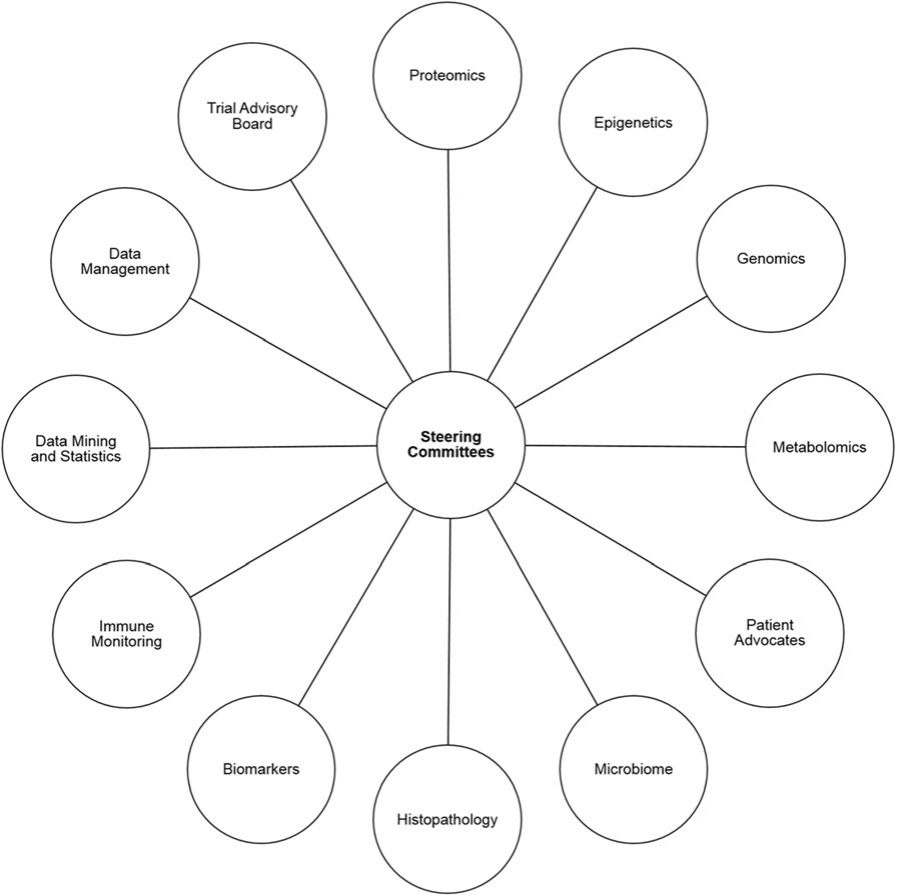
See text for description

## P720 Regional delivery of anti-CTLA-4 to induce systemic anti-tumor immunity with limited autoimmune toxicity

### Airi Harui, PhD^1^, Michael D. Roth, MD^1^, Sandra M. McLachlan, PhD^2^

#### ^1^UCLA, Los Angeles, CA, USA; ^2^Cedars-Sinai Medical Center, Los Angeles, CA, USA

##### **Correspondence:** Airi Harui (aharui@mednet.ucla.edu)

**Background**

Recent advances in immunotherapy targeting checkpoint inhibitors such as PD-1 and CTLA-4 has begun to out-perform conventional chemotherapy for melanoma, renal, lung cancer and other malignancies. While combining anti-CTLA-4 mAb (α-CTLA-4) with α-PD-1 demonstrates superior efficacy over either agent alone, this combination also magnifies the extent of inflammatory and autoimmune toxicity and can limit clinical utility. The delicate balance that exists between unleashing tumor killing and promoting autoimmune toxicity represents a major obstacle when targeting multiple immune checkpoint inhibitors. We hypothesize that regional administration of low-dose α-CTLA-4 that targets tumor-draining lymph nodes, rather than high-dose systemic therapy, may be particularly important in controlling this risk/benefit ratio.

**Methods**

In order to facilitate regional administration of α-CTLA-4 we formulated a proprietary biocompatible hydrogel that: (a) rapidly polymerizes in situ so that it can be delivered by either subcutaneous or standard image-guided needle injection, (b) protects incorporated biologic agents from degradation, (c) is optimized for the controlled delivery of mAbs over a period of 3-5 days, (d) facilitates trafficking to draining lymph nodes and (e) can be customized to self-resorb to enable repeated administration.Anti-tumor effects of regionally administered low-dose α-CTLA-4 were assessed in an MC-38 mouse model. C57BL/6 mice bearing subcutaneous tumors were treated with either hydrogel encapsulated low dose α-CTLA-4 (α-CTLA-4/hydrogel) by injection into the subcutaneous peri-tumoral tissue or conventional systemic dosing with α-CTLA-4 in PBS by intraperitoneal injection. Anti-tumor efficacy was also evaluated when regional administration of α-CTLA-4 was combined with systemic α-PD-1. The impact of treatment route on the induction of autoimmune toxicity was evaluated in a NOD-H2H4 mouse model in which administration of iodine-containing water enhances autoimmune thyroiditis. Thyroid-specific autoimmunity was assessed as the change in serum levels of anti-thyroglobulin antibody.

**Results**

Our results demonstrated 1) regional delivery of α-CTLA-4/hydrogel produced anti-tumor responses that were equal/better than systemic dosing while requiring only 1/6th of the total dosing, 2) Serum exposure to α-CTLA-4 (AUC) averaged only 1/16th of that measured following systemic dosing, 3) Regional α-CTLA-4/hydrogel synergized with systemic α-PD-1 and complete responders were immune to a secondary tumor challenge at a distant site, 4) while systemic therapy with α-CTLA-4 markedly enhanced the generation of anti-thyroglobulin antibodies in iodide-exposed NOD-H2H4 mice, regional low-dose α-CTLA-4/hydrogel had a significantly lesser effect.

**Conclusions**

Controlled regional delivery of α-CTLA-4/hydrogel has significant potential to improve the risk/benefit ratio associated with neutralizing checkpoint inhibitor therapy.

## P721 Single-cell PSI of CD8+ TILs in melanoma shows uniquely sensitive correlation with response to anti-PD-1 therapy, where histology and serum cytokines were unable to detect significant associations

### Sean G. Mackay, MBA^1^, Brianna Flynn, MS^1^, Kevin Morse^1^, Patrick Paczkowski^1^, Jonathan Chen, MS^1^, Antonella Bacchiocchi^2^, James R. Heath^3^, Rong Fan, PhD^2^, Mario Sznol, MD^2^, Ruth Halaban, PhD^2^, Jing Zhou, MD, PhD^1^

#### ^1^IsoPlexis, Branford, CT, USA; ^2^Yale Univeristy, New Haven, CT, USA; ^3^Insitute for Systems Biology, Seattle, WA, USA

##### **Correspondence:** Jing Zhou (jing@isoplexis.com)

**Background**

Functional alteration of tumor-infiltrating T lymphocytes (TILs) may serve as a predictor for clinical outcome in cancer patients receiving immunotherapy. However, due to great heterogeneity and small sample size of TILs in primary tumor tissues from cancer patients, it requires single-cell highly-multiplexed analysis for precise yet comprehensive evaluation of TILs function kinetics. In addition, increasing evidence has shown a positive correlation of polyfunctional T cells (co-secretion of 2+ proteins per single cell) with improved clinical outcome in patients after CAR-T cell therapy and vaccination. Herein, we employed a single-cell 17-plex proteomics to profile the full spectrum of TILs functionality in patients with metastatic melanoma after anti-PD-1 therapy.

**Method**

Biopsied melanoma tissues were dissociated with Collagenase I (1 mg/ml) and DNase (20 μg/ml). CD8+ TILs from the digest were enriched by CD8 microbeads, stimulated with immobilized anti-CD3 antibody (10 μg/ml) at 37°C, 5% CO2 for 24 hours and loaded into an IsoCode chip containing ~12000 microchambers pre-patterned with a complete copy of a 17-plex antibody array. After 16-hour-on-chip incubation at 37°C, 5% CO2, cytokine signals from ~2000 single cells were captured. The polyfunctional CD8+ TILs were evaluated across 4 functional groups: The polyfunctionality was evaluated across 4 functional groups: Effector (Granzyme B, IFN-gamma, MIP-1alpha, Perforin, TNF-alpha), Stimulatory (GM-CSF, IL-2, IL-5, IL-8, IL-9), Regulatory (IL-4, IL-10, IL-13, IL-22), and Inflammatory (IL-6, IL-17A, MCP-1). Melanoma tissues were also sectioned for histopathological assessment. Serum cytokines were measured with human CD8+ T cell magnetic bead panel using bead-based technology.

**Results**

The single-cell analysis of CD8+ TILs revealed a statistically significant upregulation of polyfunctional strength index (PSI) in patients who responded to anti-PD-1 antibody therapy (n=7), compared to nonresponding patients (n=4, P=0.0294, Figure 1). The enhanced PSI in responding patients was driven by antitumor associated effector proteins, including Granzyme B, IFN-gamma, TNF-alpha, MIP-1alpha and Perforin. However, cytokines in serum and histopathological metrics including TILs percentage, mitotic rate, tumor size, and necrosis, fibrosis and apoptosis measurements were unable to show any significant associations with patient outcome.

**Conclusions**

Single-cell PSI of CD8+ TILs was able to uniquely dissect the functional kinetics of CD8+ TILs from patients with metastatic melanoma and significantly distinguishes responders from nonresponders to anti-PD-1 therapy. Our study indicates the potential of PSI as an integral clinical biomarker for evaluating the efficacy of the therapy on a per-patient basis, and enables understanding of the checkpoint mechanism and its application to drug development.

**Fig. 1 (abstract P721).**
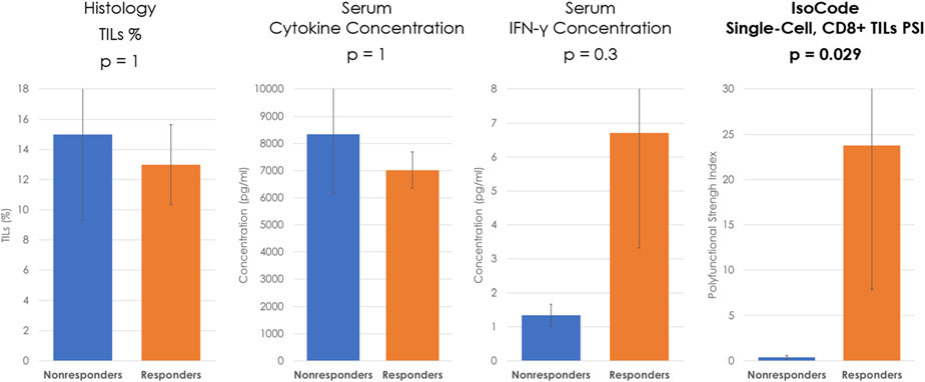
CD8+ TILs PSI Associates with Anti-PD-1 Therapy Response

